# Supplementary material for: Nef stabilizes actin to prevent HIV-1 sensing by RIG-I-like receptors
Source: Nat Commun. 2025 Dec 7;16:10945. doi: 10.1038/s41467-025-67028-5 (PMC12686415; doi:10.1038/s41467-025-67028-5)
Supplement: Supplementary file 1 — Supplementary Information [file 41467_2025_67028_MOESM1_ESM.pdf]

Supplementary Information

Nef stabilizes actin to prevent HIV-1 sensing by RIG-I-like receptors

Alexandre Laliberté[1*], Caterina Prelli Bozzo[1,2*], Dhiraj Acharya[3], Aurora De Luna[1], Maximilian Hirschenberger[1], Junji Zhu[3], Meta Volcic[1], Bettina Stolp[4], Cristina Rodriguez[4], Oliver T. Fackler[4,5], Michaela U. Gack[3], Konstantin M.J. Sparrer[1,6#], Frank Kirchhoff[1#]

[1]Institute of Molecular Virology, Ulm University Medical Center, Ulm, Germany. [2]Department of Microbial Pathogenesis, Yale University School of Medicine, New Haven, CT, USA. [3]Florida Research and Innovation Center, Cleveland Clinic, Port Saint Lucie, USA. [4]Department of Infectious Diseases, Integrative Virology, CIID, Heidelberg University, Medical Faculty Heidelberg, Heidelberg, Germany. [5]German Centre for Infection Research (DZIF), Partner Site Heidelberg, Germany. [6]German Center for Neurodegenerative Diseases (DZNE), Ulm, Germany.

*Both contributed equally

# Corresponding authors: Konstantin.Sparrer@uni-ulm.de and Frank.Kirchhoff@uni-ulm.de

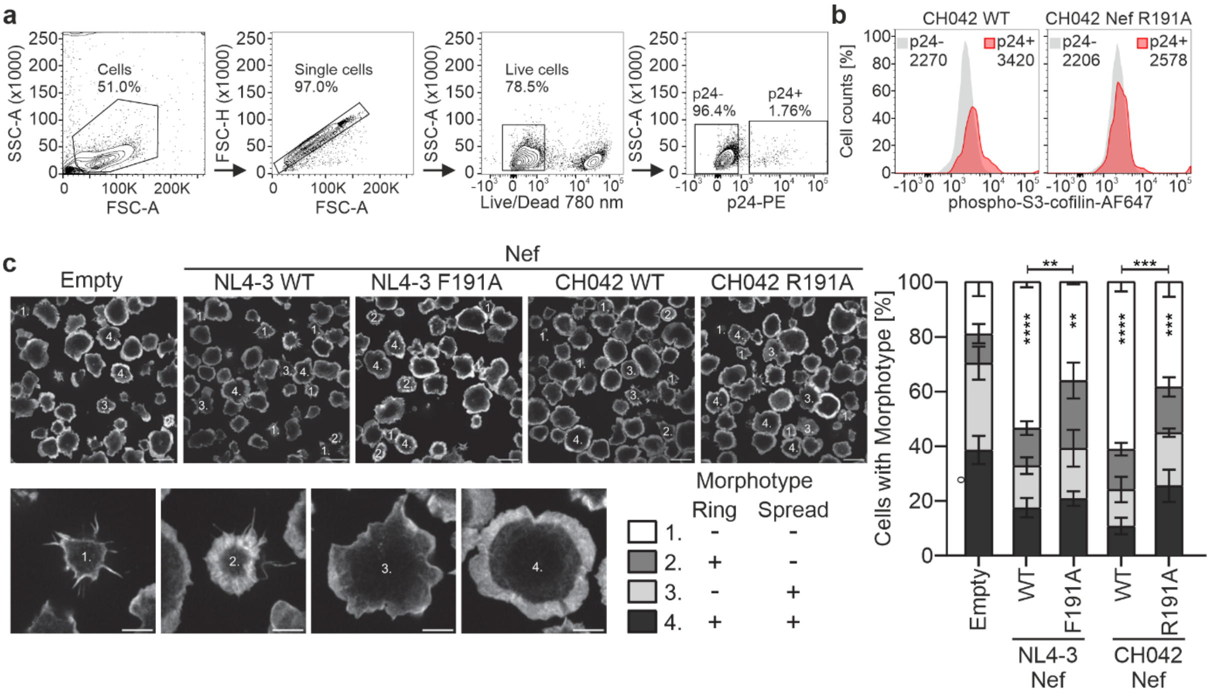

Supplementary Fig. 1 Effects of Nef variants on cofilin phosphorylation as well as cell spreading and actin polymerization in response to T cell activation. a-b, Gating strategy (a) and histogram (b) showing pS3-cofilin signals in PBMCs from one donor in infected (p24+) cells (red) vs uninfected (p24-) cells (grey) for CH042 WT (left) or R191A Nef (right). Numbers indicate media fluorescence intensity of pS3-cofilin. **c,** Effect of Nef on the morphological responsne of CD4 T cells to surface-mediated TCR stimulation. JTAg cells were co-transfected with pEGFP and pTWIST plasmids as control or with the pEGFP expression plasmid and a pTWIST-based expression plasmid for the indicated Nef variant. 24 hours post transfection, cells were exposed to TCR-stimulatory surfaces and analyzed for their morphological and actin remodeling response. Upper panel: Representative confocal micrographs in the F-actin channel (phalloidin-TRITC). White asterisks indicate transfected, GFP-positive cells. Scale bar: 15 µm. Lower panel: Representative micrographs of the four cellular morphotypes observed according to Imle et al., 2015 mBio: 1. No F-actin ring, no spreading, 2. F-actin ring but no spreading, 3. No F-actin ring but spreading, 4. F-actin ring and spreading. Scale bar: 10 µm. Histogram: Quantification of the distribution of cells into four morphotypes. Results represent mean (±SD) from three independent experiments with at least 100 cells analyzed per condition each. Statistical analysis to the GFP control or between a Nef mutant and its corresponding wild type was performed using Ordinary one-way-Anova with Dunnett correction. **, P< 0.01; ***, P< 0.001. Exact P values and Source data are provided in the Source data file.

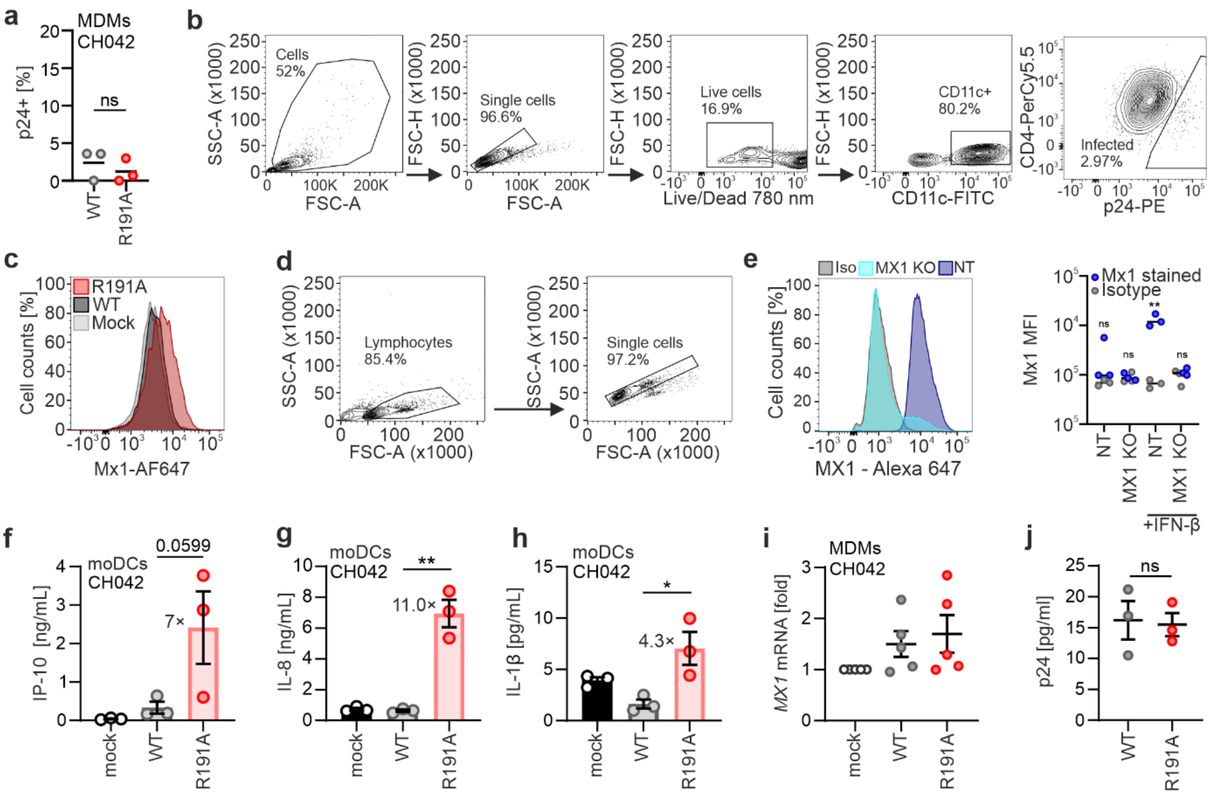

Supplementary Fig. 2. Immune activation in MDMs and moDCs. a, Percentage of p24+ in cells infected in Fig. 1a, measured by flow cytometry. Data shows mean (±SEM), each point represents one donor (N=3 donors). **b-c,** Gating strategy (b) and histogram (c) showing Mx1 levels in MDMs infected with VSV-G pseudotyped CH042 WT (black) or R191A Nef (red) at 48 hours post infection, measured by flow cytometry. **d-e,** Gating strategy (d), histogram (e, left), and quantification (e, right) showing Mx1 levels or isotype control (iso; grey) signal measured by flow cytometry in $CD4^+$ T cells electroporated with Cas9/NT-sgRNA (dark blue) or Cas9/*MX1*-sgRNA (light blue) complex and treated with 100 U/mL IFN-β for 24 hours. Data shows mean, each point represents one donor (N=3 donors). **f-h,** IP10 (g), IL-8 (g) and IL-1β (h) levels in supernatants of monocyte-derived dendritic cells (moDCs) infected as in Fig. 1a. Each point represents the mean of one donor. Bars represent mean of the donors (±SEM, N=3 donors). **i,** qRT-PCR analysis of cellular *MX1* mRNA levels in primary MDMs infected with non-pseudotyped CH042 expressing WT Nef (grey) or Nef R191A (red) at 4 days post infection (N=5 donors). Each point represents the mean of one donor. **j,** Levels of p24 in the supernatants of MDMs infected as in i. Statistical analysis was done using two-way ratio paired t tests. P values are indicated as *p < 0.05; **p < 0.01 or not significant (ns, p > 0.05). Exact P values and Source data are provided in the Source data file.

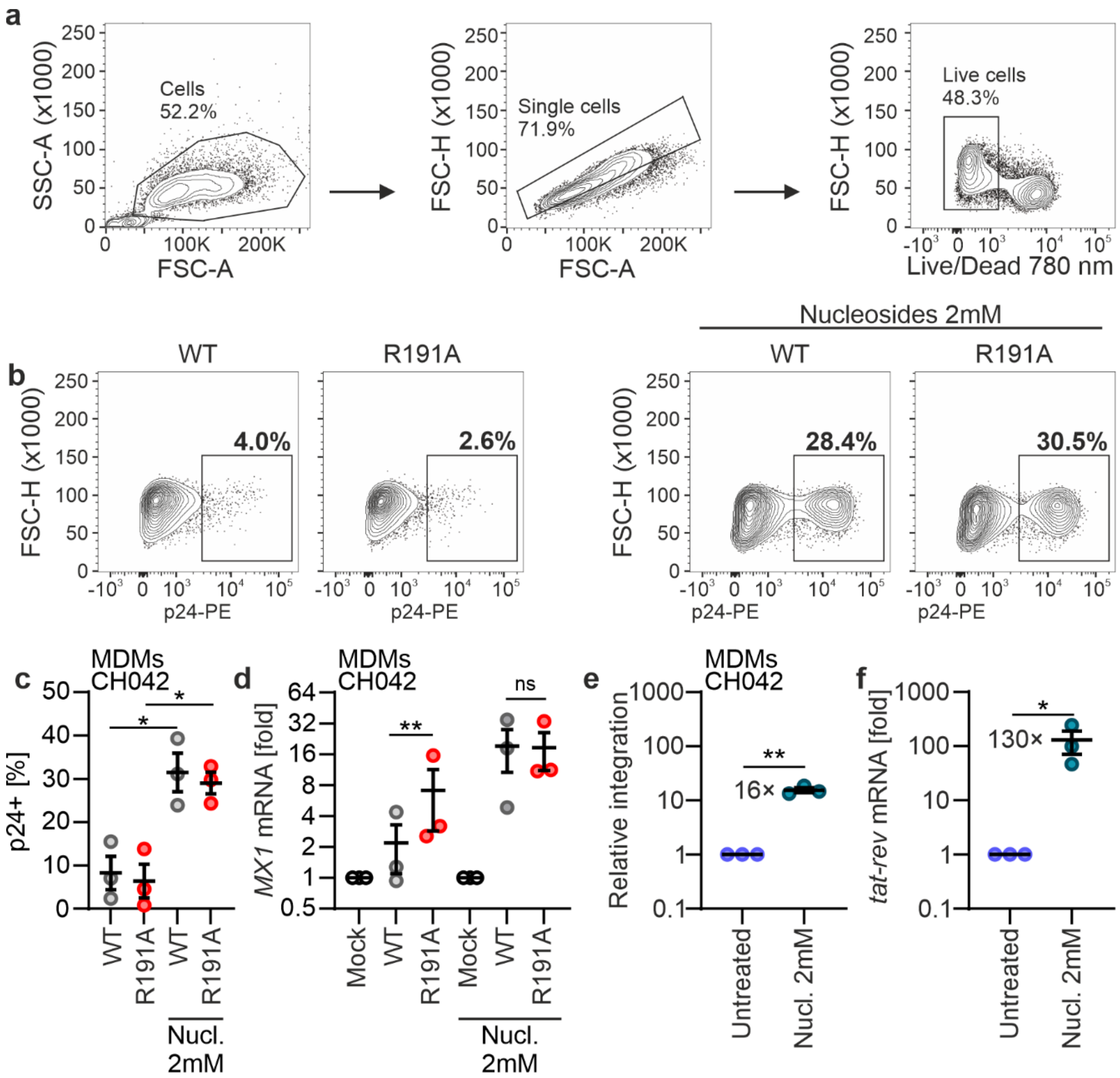

Supplementary Fig. 3. Nucleosides-assisted infection of MDMs. a, Gating strategy used to analyze infection in primary MDMs by flow cytometry. **b,** Representative flow cytometry data showing MDMs infected with VSV-G pseudotyped CH042 WT or R191A Nef in absence or presence of 2 mM nucleosides, at 48 hours post infection. **c,** Quantification of (b). Data shows mean (±SEM), each point (WT, grey; R1919A, red) represents one donor (N=3 donors). Statistical analysis was done using two-way Welch's t-test. **d,** qRT-PCR analysis of cellular *MX1* mRNA levels in primary MDMs infected as in (a) in absence or presence of 2 mM nucleosides. Data shows mean (±SEM) relative to mock uninfected cells, each point represents the mean of one donor measured in duplicates (N=3 donors) **e,** Relative proviral integration measured in DNA of MDMs infected with VSV-G pseudotyped CH042 WT in absence (blue) or in presence (petrol) of 2 mM nucleosides, at 48 hours post infection. Data shows mean (±SEM) normalized to values obtained in absence of nucleosides, each point represents the mean of one donor measured in duplicates (N=3 donors). **f,** qRT-PCR analysis of HIV *tat-rev* spliced HIV RNA in infected primary MDMs from (e). Data shows mean (±SEM) normalized to values obtained in absence of nucleosides, each point represents the mean of one donor measured in duplicates (N=3 donors). Statistical analysis was done using two-way ratio paired t tests. P values are indicated as *$p < 0.05$, **$p < 0.01$ or not significant (ns, $p > 0.05$). Exact P values and Source data are provided in the Source data file.

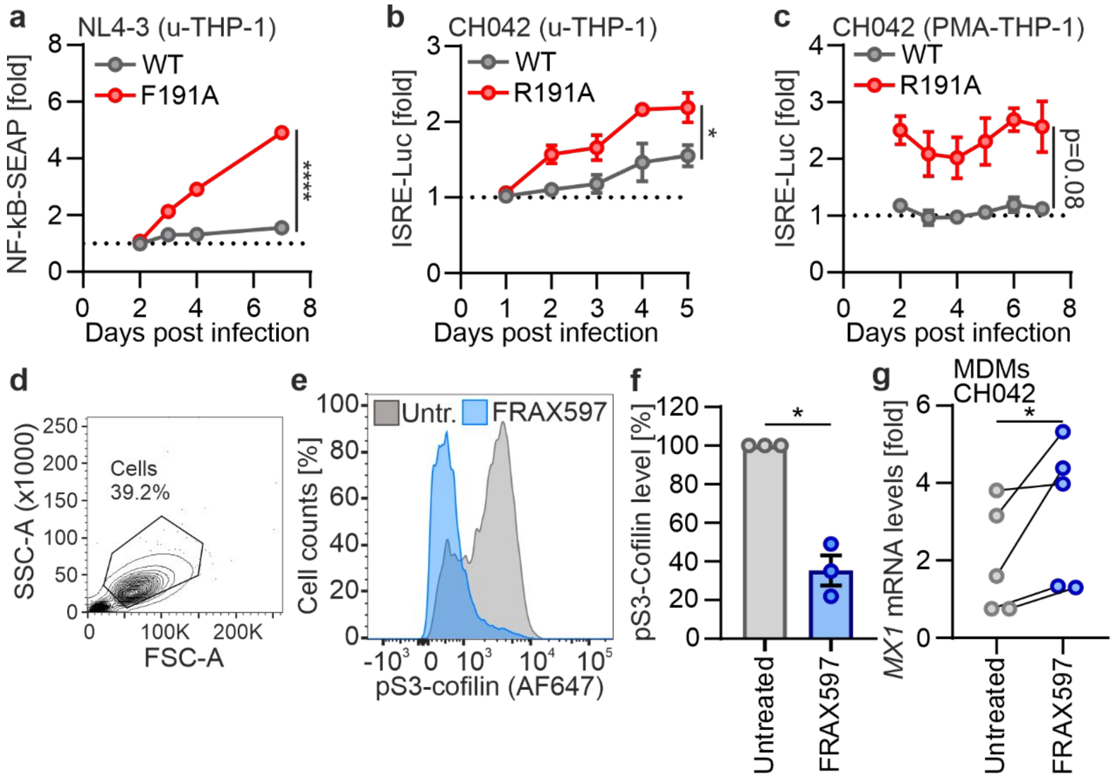

Supplementary Fig. 4 PAK2 is involved in immune sensing. a, Relative NF-κB induction measured by secreted alkaline phosphatase (SEAP) in THP-1 duals cells containing ISRE-Luciferase and NFκB reporter genes infected with VSV-G pseudotyped NL4-3 expressing WT (grey) or F191A (red) Nef. Each point represents the mean (±SEM) of triplicates. Statistical analysis was done using repeated-measure two-way ANOVA. **b-c,** Relative luciferase (Luc) induction in THP-1 duals cells either undifferentiated (b) or PMA-differentiated (c) infected with VSV-G pseudotyped CH042 WT (grey) or expressing R191A (red) Nef. Each point represents the mean (±SEM) of triplicates. Statistical analysis was done using repeated-measure two-way ANOVA, Geisser-Greenhouse's correction. **d,** Example of gating for MDMs treated with FRAX597. **e,** Representative histogram for phospho-S3-cofilin analysis in MDMs treated with 25 µM FRAX597 (blue). **f,** Quantification of median fluorescence in (e) normalized to untreated cells (grey). Bars represent mean (±SEM) and points represent donors (N=3 donors). Statistical analysis was done using two-way Welch's t test. **g,** qRT-PCR analysis of *MX1* mRNA induction in primary MDMs infected with VSV-G pseudotyped HIV-1 CH042 WT 48 hours post-infection in absence (grey) presence (blue) of 1 µM FRAX597. Values relative to uninfected cells. Bars represent mean (±SEM), points represent donors (N=5 donors). Unless otherwise indicated statistical analysis was done using two-way ratio paired t tests. P values are indicated as $^*p < 0.05$; $^{**}p < 0.01$, $^{****}p < 0.0001$ or not significant (ns, $p > 0.05$). Exact P values and Source data are provided in the Source data file.

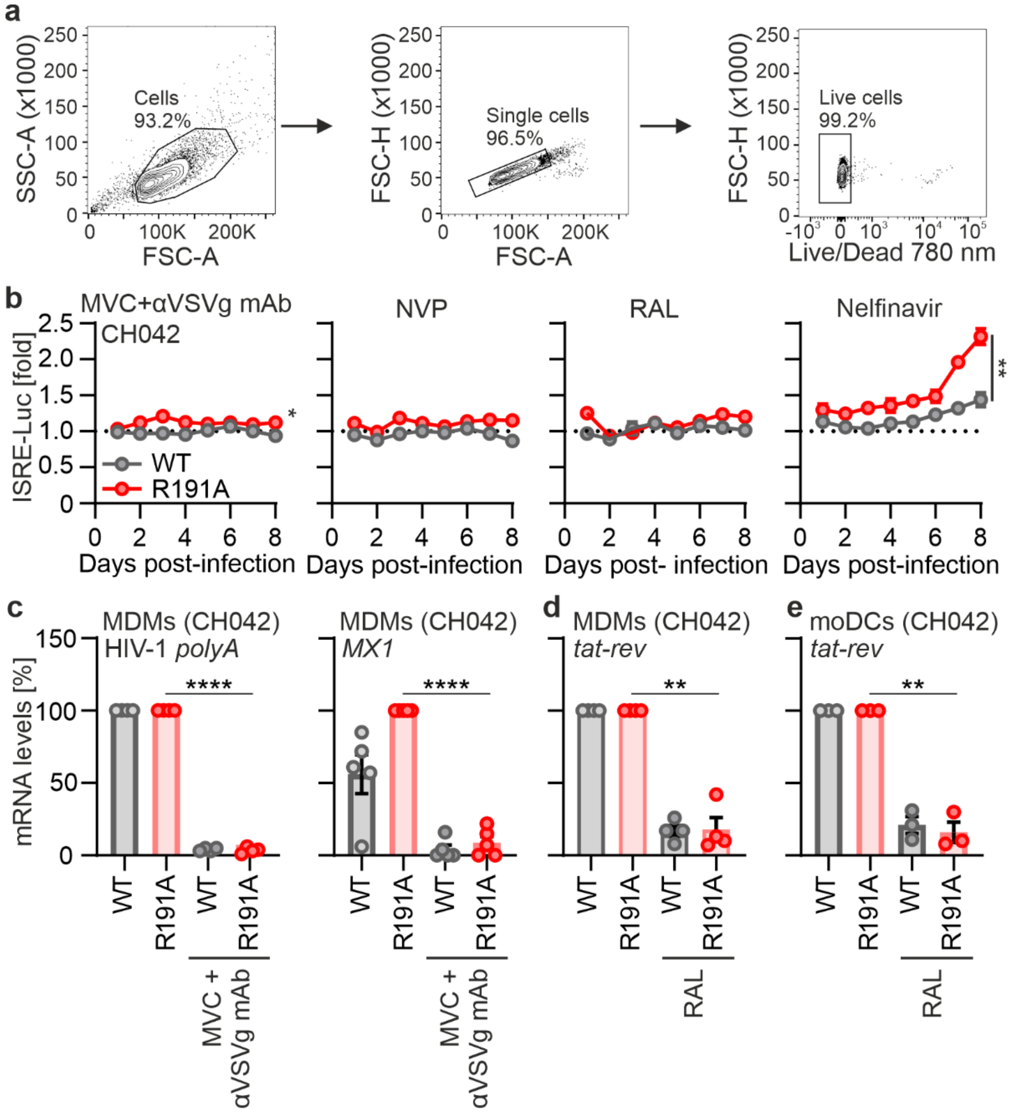

Supplementary Fig. 5 Nef counteracts sensing of a post-integration step. a, Gating strategy for figure 3a. Cells are pre-gated on live cells **b,** Relative luciferase induction in THP-1 duals reporter cells infected with VSV-G pseudotyped HIV-1 CH042 WT (grey) or expressing Nef R191A (red) and treated with Maraviroc (MVC) and anti-VSVg neutralizing antibodies, Nevirapine (NVP), Raltegravir (RAL) or Nelfinavir (NFV). Each point represents the mean of triplicates. Repeated-measure two-way ANOVA, Geisser-Greenhouse's correction. **c,** qRT-PCR analysis of polyA HIV-1 RNA (left) and *MX1* mRNA induction (right) in primary MDMs infected with VSV-G pseudotyped HIV-1 CH042 WT (grey) or Nef R191A (red) at 48 hours post-infection in presence of Maraviroc (MVC) and anti-VSVg neutralizing antibodies. For polyA HIV-1 RNA, values are relative to untreated cells, for *MX1,* values are relative to R191A-infected, untreated cells. Each point represents the mean of one donor measured in duplicates. Bars represent the mean (±SEM) of donors (N=4 donors, polyA; N=5 donors, *MX1*). **d-e,** qRT-PCR analysis of HIV *tat-rev* spliced HIV RNA in primary MDMs (d) or moDCs (e) infected with VSV-G pseudotyped HIV-1 CH042 WT (grey) or Nef R191A (red) at 48 hours post-infection in presence of Raltegravir (RAL). Values relative to corresponding untreated cells. Each point represents the mean of one donor measured in duplicates. Bars represent the mean (±SEM) of donors (N=4 donors). Unless otherwise indicated statistical analysis was done using two-way Welch's t tests. p values are indicated as $**p < 0.01$, $****p < 0.0001$. Exact P values and Source data are provided in the Source data file.

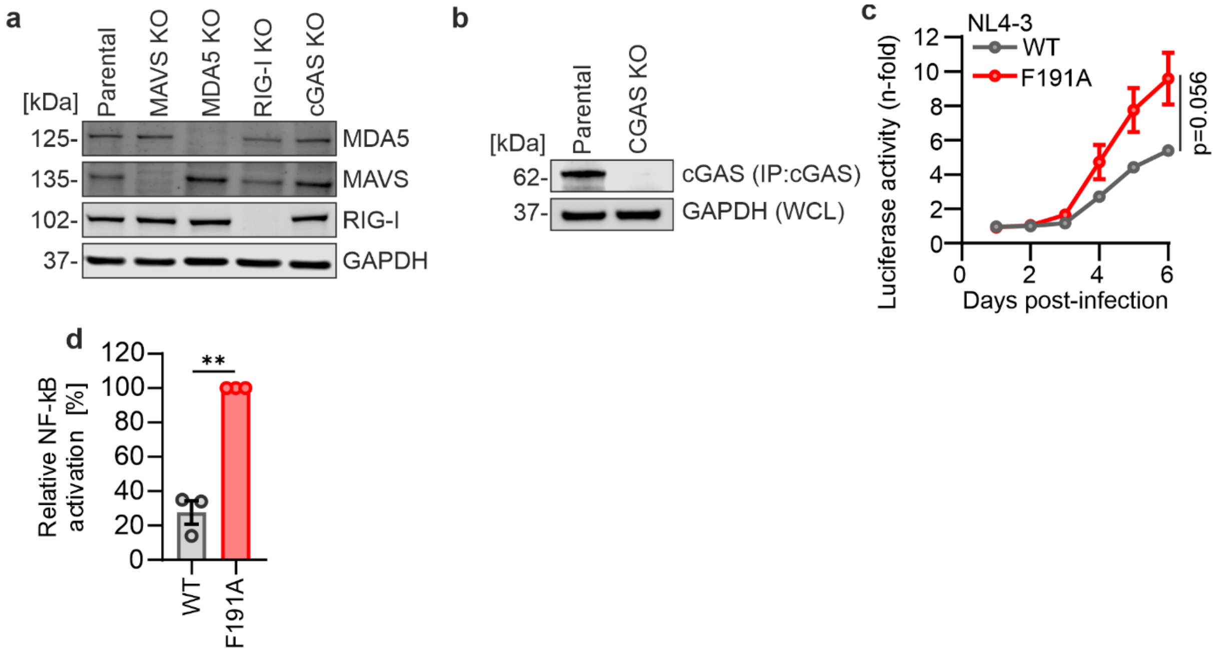

Supplementary Fig. 6. Induction of ISRE and NF-κB in parental THP-1 and KO validation. a, Western blot showing the expression of endogenous MDA5, MAVS and RIG-I in the parental cells and in *MAVS* KO, *MDA5* KO, *RIG-I* KO and *CGAS* KO THP-1 dual cells. **b,** Western blot showing the expression of endogenous cGAS after cGAS immunoprecipitation in parental THP-1 dual cells and *CGAS* KO cells. **c,** Relative luciferase induction in THP-1 duals cells containing ISRE-Luciferase and NF-κB reporter genes infected with VSV-G pseudotyped NL4-3 expressing WT (grey) or F191A Nef (red). Each point shows the mean (±SEM) of triplicates. Statistical analysis was done using repeated measure two-way ANOVA, Geisser-Greenhouse's correction. **d,** Relative NF-κB induction in THP-1 dual cells infected with VSV-G pseudotyped NL4-3 expressing WT (grey) or F191A Nef (red), measured at 96 hours post infection. Bars show mean (±SEM), each point represent the mean of three experiments. Statistical analysis was done using Welch's t test. p values are indicated as $^{**}p < 0.01$, or not significant ($p > 0.05$). Exact P values and Source data are provided in the Source data file.

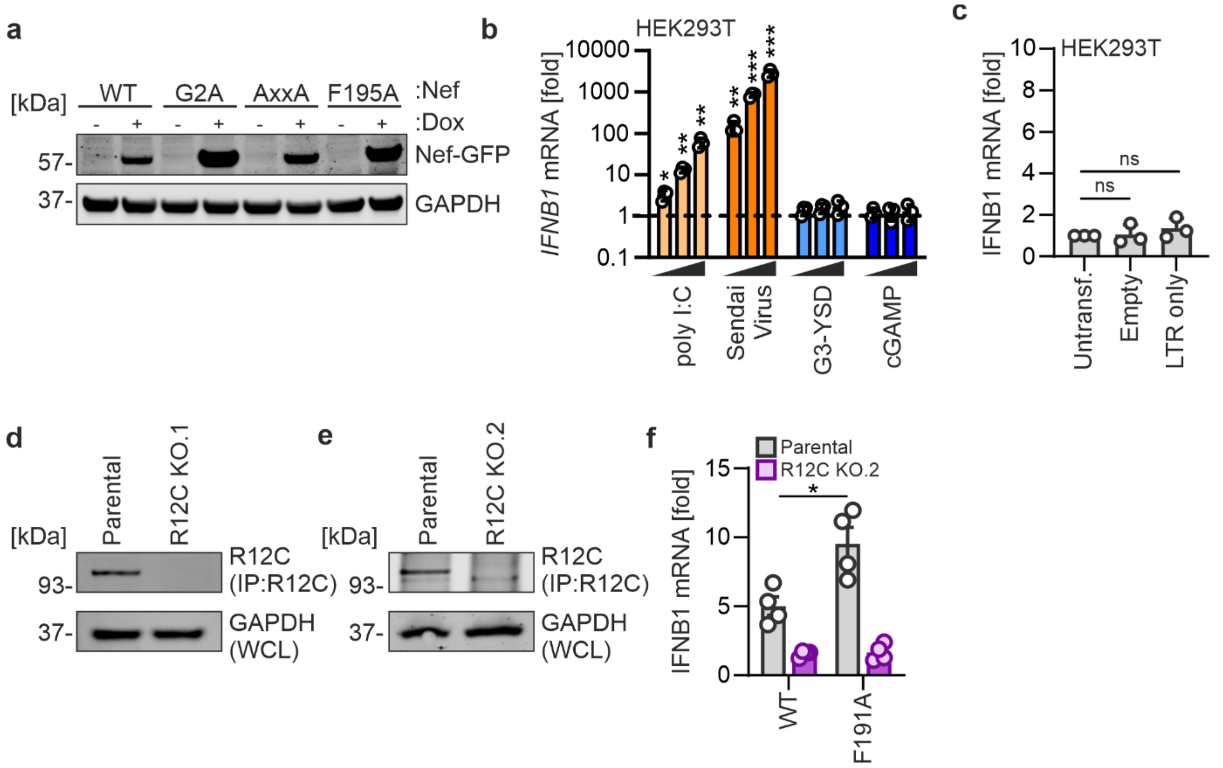

Supplementary Fig. 7. Nef inhibits sensing by RLRs and activation of MDA5. a, Western blot showing Nef expression in Dox inducible CHO cells expressing the indicated Nef WT or mutants in presence or absence of doxycycline (Dox) after 48 hours of treatment. **b,** qRT-PCR analysis of *IFNB* mRNA in HEK293T cells that were either transfected with poly (I:C) HMW (light orange), infected with Sendai virus (dark orange), transfected with G3-YSD (light blue) or treated with cGAMP (dark blue) at 24 h post treatment. Each point represents the mean (±SEM) of triplicates. Statistical analysis was done using two-way ratio paired t test. **c,** qRT-PCR analysis of *IFNB1* mRNA in HEK293T that were left non-transfected or were transfected with empty empty vector or a vector expressing the HIV-1 LTR at 48 h post transfection. Bars represent the mean (±SEM) of three experiments**.** Statistical analysis was done using two-way ratio paired t test **d-e,** Western blot showingthe expression of R12C after R12C immunoprecipitation in parental ells and *R12C* KO.1 cells (d) or *R12C* KO.2 cells (e). **f,** qRT-PCR of cellular *IFNB1* mRNA levels in parental or *R12C* KO.2 HEK293T cells transfected with proviral HIV-1 NL4-3 expressing WT (grey) or F191A (purple) Nef. Bars show mean (±SEM) relative to cells transfected with an empty vector. (N=4 independent experiments). Statistical analysis was done using two-way Welch's t test. p values are indicated as *$p < 0.05$; **$p < 0.01$; ***$p < 0.001$; ****$p < 0.0001$ or not significant ($p > 0.05$). Exact P values and Source data are provided in the Source data file.

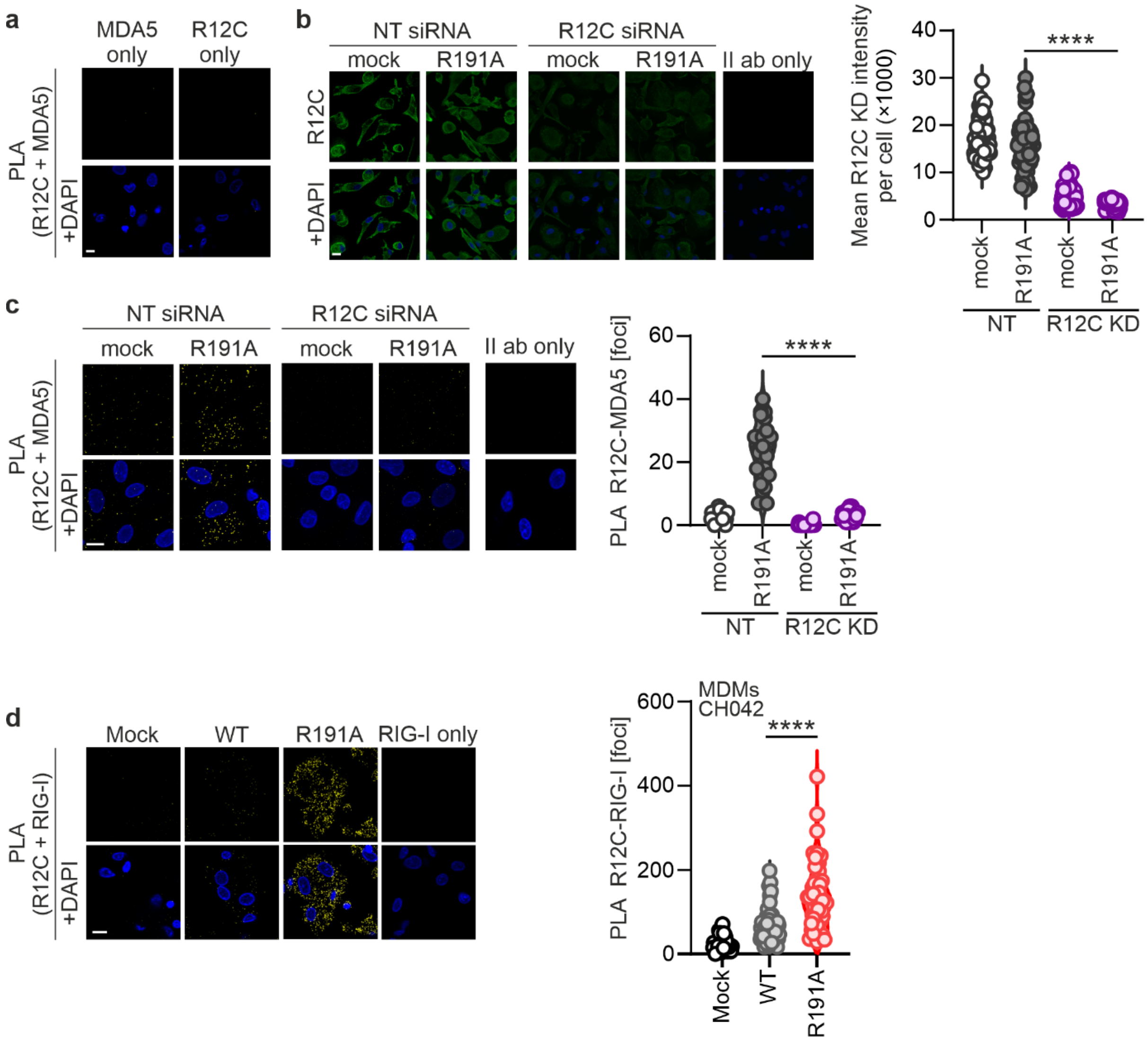

Supplementary Fig. 8. Nef prevents relocalization of R12C. a, Single antibody controls of proximity ligation assay (PLA) in Fig. 5d in infected MDMs. **b,** Representative confocal microscopy images showing R12C signal intensity (green) and DAPI (blue) in primary MDMs after NT siRNA (grey) control or knock-down of R12C (purple) and either left uninfected or infected with VSV-G pseudotyped HIV-1 expressing R191A Nef in presence of 0.5 mM nucleosides. Quantification of mean R12C signal intensity per cell (N=68 cells, mock; N=56 cells, R191A; N=58 cells, mock; N=79 cells, R191A). **c,** PLA of endogenous R12C and MDA5 in primary MDMs treated with NT control siRNA or R12C siRNA as in b. Quantification of the PLA signal (right). Each point represents the number of foci per cell (N=22 cells, mock; N=25 cells, R191A; N=20 cells, mock; N=21 cells, R191A). **d,** PLA (left) of endogenous R12C and RIG-I in primary MDMs infected as described with VSV-G pseudotyped CH042 WT (grey) or R191A Nef (red) at 48 h post infection and quantification (right). Each point represents the number of foci per cell (N=50 cells). Statistical analysis was done using two-way Welch's t test. P values are indicated as ****$p < 0.0001$). Exact P values and Source data are provided in the Source data file.

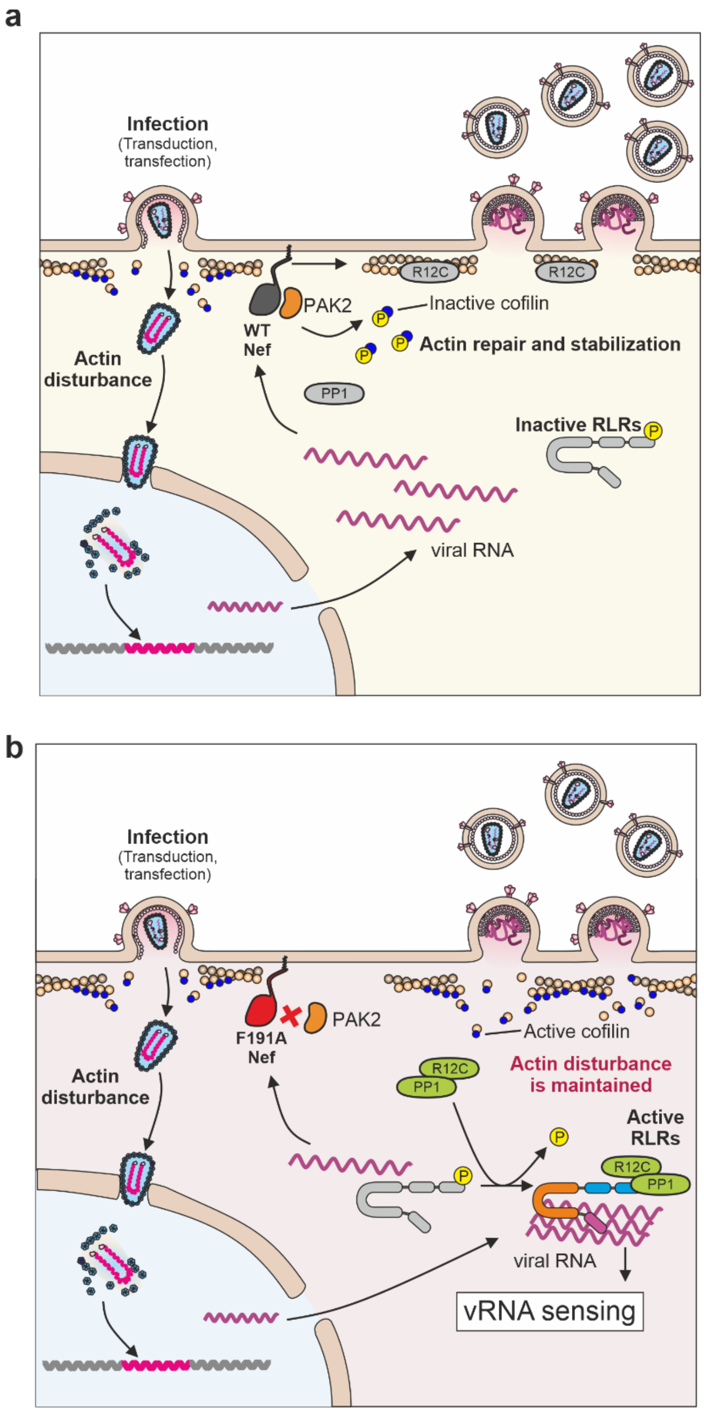

Supplementary Fig. 9. Schematic model for Nef-mediated inhibition of HIV-1 post-integration sensing by RLRs. Genuine and VSV-G pseudotyped HIV-1 infection, or transfection of plasmids all trigger filamentous actin cytoskeleton disturbance. **a,** The accessory HIV-1 protein Nef is abundantly expressed after proviral integration, and interacts with PAK2 to inactivate cofilin, which promotes actin depolymerization and turnover. This allows repair and stabilization of actin filaments and prevents the release of the phosphatase complex comprising protein phosphatase 1 (PP1) and the PP1 regulatory subunit R12C (R12C), which is critical for the activation of RIG-I-like receptors (MDA5/RIG-I). Thus, Nef suppresses post-integration sensing of viral RNA (vRNA) transcripts. **b,** The F/R191A mutation impairs Nef binding to PAK2. Consequently, actin disturbances are maintained and the PP1-R12C complex mediates dephosphorylation and activation of RLRs, resulting in RLR activation and heightened interferon and ISG response in immune cells.
