# Supplementary material for: Nef stabilizes actin to prevent HIV-1 sensing by RIG-I-like receptors
Source: Nat Commun. 2025 Dec 7;16:10945. doi: 10.1038/s41467-025-67028-5 (PMC12686415; doi:10.1038/s41467-025-67028-5)
Supplement: Supplementary file 2 — Description Of Additional Supplementary File [file 41467_2025_67028_MOESM2_ESM.pdf]

Description of additional supplementary files

Supplementary data 1

Description: List of primers
