# Supplementary material for: Nef stabilizes actin to prevent HIV-1 sensing by RIG-I-like receptors
Source: Nat Commun. 2025 Dec 7;16:10945. doi: 10.1038/s41467-025-67028-5 (PMC12686415; doi:10.1038/s41467-025-67028-5)
Supplement: Supplementary file 4 — Reporting Summary [file 41467_2025_67028_MOESM4_ESM.pdf]

nature portfolio

Corresponding author(s): Frank Kirchhoff, ORCID: 0000-0002-7052-2360
Konstantin M.J. Sparrer, ORCID: 0000-0002-8682-1779

Last updated by author(s): 13.11.2025

Reporting Summary

Nature Portfolio wishes to improve the reproducibility of the work that we publish. This form provides structure for consistency and transparency in reporting. For further information on Nature Portfolio policies, see our Editorial Policies and the Editorial Policy Checklist.

Statistics

For all statistical analyses, confirm that the following items are present in the figure legend, table legend, main text, or Methods section.

n/a	Confirmed	
☐	☒	The exact sample size (*n*) for each experimental group/condition, given as a discrete number and unit of measurement
☐	☒	A statement on whether measurements were taken from distinct samples or whether the same sample was measured repeatedly
☐	☒	The statistical test(s) used AND whether they are one- or two-sided<br>*Only common tests should be described solely by name; describe more complex techniques in the Methods section.*
☒	☐	A description of all covariates tested
☒	☐	A description of any assumptions or corrections, such as tests of normality and adjustment for multiple comparisons
☐	☒	A full description of the statistical parameters including central tendency (e.g. means) or other basic estimates (e.g. regression coefficient) AND variation (e.g. standard deviation) or associated estimates of uncertainty (e.g. confidence intervals)
☐	☒	For null hypothesis testing, the test statistic (e.g. *F*, *t*, *r*) with confidence intervals, effect sizes, degrees of freedom and *P* value noted<br>*Give P values as exact values whenever suitable.*
☒	☐	For Bayesian analysis, information on the choice of priors and Markov chain Monte Carlo settings
☒	☐	For hierarchical and complex designs, identification of the appropriate level for tests and full reporting of outcomes
☐	☒	Estimates of effect sizes (e.g. Cohen's *d*, Pearson's *r*), indicating how they were calculated

Our web collection on statistics for biologists contains articles on many of the points above.

Software and code

Policy information about availability of computer code

Data collection	BD FACSDiva™ Version 8.0, LI-COR Image Studio Version 5.2, StepOnePlus Real-Time PCR System software, 2019 Qognit, Inc. Version Version 2024-06-15, LSM710 confocal microscope Zeiss
Data analysis	GraphPad Prism Version 10, Corel DRAW 23.1, LI-COR Image Studio Lite Version 5.0.21,, FlowJo 10.8.1, Fiji 2.3.0 ImageJ

For manuscripts utilizing custom algorithms or software that are central to the research but not yet described in published literature, software must be made available to editors and reviewers. We strongly encourage code deposition in a community repository (e.g. GitHub). See the Nature Portfolio guidelines for submitting code & software for further information.

Data

Policy information about availability of data

All manuscripts must include a data availability statement. This statement should provide the following information, where applicable:

- Accession codes, unique identifiers, or web links for publicly available datasets
- A description of any restrictions on data availability
- For clinical datasets or third party data, please ensure that the statement adheres to our policy

A data availability statement is included, Raw data for e.g. Western blots are provided in the source data file

Research involving human participants, their data, or biological material

Policy information about studies with human participants or human data. See also policy information about sex, gender (identity/presentation), and sexual orientation and race, ethnicity and racism.

Reporting on sex and gender	N.A.
Reporting on race, ethnicity, or other socially relevant groupings	N.A.
Population characteristics	N.A.
Recruitment	N.A.
Ethics oversight	N.A.

Note that full information on the approval of the study protocol must also be provided in the manuscript.

Field-specific reporting

Please select the one below that is the best fit for your research. If you are not sure, read the appropriate sections before making your selection.

☒ Life sciences ☐ Behavioural & social sciences ☐ Ecological, evolutionary & environmental sciences

For a reference copy of the document with all sections, see nature.com/documents/nr-reporting-summary-flat.pdf

Life sciences study design

All studies must disclose on these points even when the disclosure is negative.

Sample size	No sample size calculations were performed. Experiments were perfomed at least in triplicates and with multiple donors as stated.
Data exclusions	no data was excluded
Replication	The number of independent replicates to similar results is indicated in the respective figure legends or the Statistics and Reproducibility section to keep the main figure legends concise. All technically sound attempts at replication yielded similar results.
Randomization	Randomization was not applicable for this study, as no human trials or cohort studies were performed or samples assigned to experimental groups.
Blinding	Blinding was not applicable.

Reporting for specific materials, systems and methods

We require information from authors about some types of materials, experimental systems and methods used in many studies. Here, indicate whether each material, system or method listed is relevant to your study. If you are not sure if a list item applies to your research, read the appropriate section before selecting a response.

Materials & experimental systems

n/a	Involved in the study	
☐	☒	Antibodies
☐	☒	Eukaryotic cell lines
☒	☐	Palaeontology and archaeology
☒	☐	Animals and other organisms
☒	☐	Clinical data
☒	☐	Dual use research of concern
☒	☐	Plants

Methods

n/a	Involved in the study	
☒	☐	ChIP-seq
☐	☒	Flow cytometry
☒	☐	MRI-based neuroimaging

Antibodies

Antibodies used	Anti MxA – Alexa Fluor 647Abcam Cat# ab237299 (1:500)<br>Anti p24 – FITC Beckman Coulter Cat# 6604665 (1:100)<br>Anti p24 – RD1(PE) Beckman Coulter Cat# 6604667 (1:100)

Anti-AU1 (rb) Novus Cat# NB600-453
Anti-actin Santa Cruz clone C4 (1:1000)
Anti-V5 Novex #R960-25 (1:5000)
Anti-CD4 – PerCP-Cy5.5 Biolegend Cat# 317428 (1:50)
Anti-GAPDH (rat) Biolegend Cat# 607902 (1:1000)
Anti-FLAG (ms) Sigma-Aldrich clone M2 (1:2000)
Anti-ISG15 (ms) Santa Cruz Biotechnogy Cat# sc-166755 (1:500)
Anti-MAVS (rb)Cell Signaling Technologies Cat# 3993 (1:1000)
Anti-MDA5 (rb) (For IF, IP)Proteintech Cat# 21775-1-AP (1:50)
Anti-MDA5 (rb) (for WB)Cell Signaling Technologies Cat# 5321 Cat# (1:1000)
Anti-Pak2 (rabbit)Cell Signaling Technologies Cat# 2608 (1:500)
Anti-PPP1R12C (ms) Santa Cruz Biotechnogy Cat# sc-398415 (1:10)
Anti-PPP1R12C (rb)Kindly provided by Prof. Dr. Michaela Gack110 (1:50)
Anti-pS3-Cofilin (rabbit)Cell Signaling Technologies Cat# 3313 (1:500)
Anti-pS88-MDA5 Kindly provided by Prof. Dr. Michaela Gack199 (1:200)
Anti-RIG-I (ms)Adipogen Cat# AG-20B-0009-C100,clone ALME-I (1:50)
Anti-human CD11c FITC. Biolegend Cat# 301603, clone 3.9 (1:20)
Anti-cGAS (rb) polyclonal antibody. Proteintech. Cat- 26416-1-AP
RDye® 680RD Goat anti-Rabbit IgG (H + L) LI-COR Cat# 926-68071 (1:20000);
IRDye® 800CW Goat anti-Mouse IgG (H + L) LI-COR Cat# 926-32210 (1:20000);
IRDye® 800CW Goat anti-Rabbit IgG (H + L) LI-COR Cat# 926-32211(1:20000);
IRDye 800RD Goat anti-Rat IgG (H + L) LI-COR Cat# 925-32219 (1:20000);
IRDye 680RD Goat anti-Rat IgG (H + L) LI-COR Cat# 926-68071 (1:20000)

Validation

Abcam:
Antibody Validation for Western blot
Antibodies are validated in western blot using lysates from cells or tissues that we have identified to express the protein of interest. Once we have determined the right lysates to use, western blots are run and the band size is checked for the expected molecular weight. We will always run several controls in the same western blot experiment, including positive lysate and negative lysate.
When possible, we also include knock-out (KO) cell lines as a true negative control for our western blots. We are always increasing the number of KO-validated antibodies we provide. In addition, we run old stock alongside our new stock. If we know the old stock works well, this also acts as a suitable positive control.
If the western blot result gives a clear clean band and we are happy with the result from the control lanes, these antibodies will be passed and added to the catalog.
Cell Signalling:
Antibody Validation for Immunofluorescence:
Cell lines or tissues with known target expression levels are used to verify specificity.
Appropriate cell lines and tissues are used to verify subcellular localization.
Antibody performance is assessed on appropriate tissues.
Cells are subjected to phosphatase treatment to verify phospho-specificity. Target specificity is also verified with the use of known knockout or null cell lines.
For Mx1 antibody, we tested in KO cells.
Cells are subjected to siRNA treatment or over-expression of the target protein to verify target specificity.
Activation state specification, target expression, and translocation are examined using ligands or inhibitors to modulate pathway activity.
Requirement of threshold signal-to-noise ratio in antibody:isotype comparison and minimum fold-induction for phospho-specific antibodies ensures the greatest possible sensitivity.
Fixation and permeabilization conditions are optimized; alternative protocols are recommended if necessary.
Stringent testing ensures lot-to-lot consistency.
Antibody Validation for Western Blotting:
Examination of several cell lines and/or tissues of known expression levels allows accurate determination of species cross-reactivity and verifies specificity.
Treatment of cell lines with growth factors, chemical activators or inhibitors, which induce or inhibit target expression, verifies specificity. Phosphatase treatment confirms phospho-specificity.
The use of siRNA transfection or knockout cell lines verifies target specificity.
Side-by-side comparison of lots to ensures lot-to-lot consistency.
Optimal dilutions and buffers are predetermined, positive and negative cell extracts are specified, and detailed protocols are already optimized, saving valuable time and reagents.

Bio Legend:
As knocking out the target protein is one of the most trusted antibody validation processes, we are starting to validate our Cell Biology portfolio antibodies by KO (knockout) and KD (knockdown) systems. To confirm antibody specificity, Western blot data using BioLegend's in-house generated CRISPR/Cas9 and siRNA, as well as CRISPR/Cas9 KO cell lysates from a collaboration with EdiGene (a genome editing company) will be made readily available to researchers
Proteintech:
Every antibody is extensively validated by an in-house team of scientists using unmodified samples and endogenous levels of proteins. With the increasing need for antibodies with high specificity and reproducibility, Proteintech introduced siRNA knockdown validation, one of the most trusted and accepted forms of antibody validation. Look out for the siRNA Knockdown symbol across the Proteintech catalog.

Eukaryotic cell lines

Policy information about cell lines and Sex and Gender in Research

Cell line source(s)	CHO.Nef-GFP AxxA Chinese hamster ovary cells expressing SF2 Nef PxxP>AxxA Prof. Dr. Oliver Fackler (Heidelberg, Germany)<br>CHO.Nef-GFP F195A Chinese hamster ovary cells expressing SF2 Nef F195A _GFP Prof. Dr. Oliver Fackler (Heidelberg, Germany)<br>CHO.Nef-GFP G2A Chinese hamster ovary cells expressing SF2 Nef G2A _GFP Prof. Dr. Oliver Fackler (Heidelberg, Germany)<br>CHO.Nef-GFP WT Chinese hamster ovary cells expressing SF2 Nef _ GFP KProf. Dr. Oliver Fackler (Heidelberg, Germany)<br>Human HEK293T cells ATCC Cat# CRL-3216<br>HEK293T R12C KO provided by Prof. Dr. Michaela Gack (Port Saint Lucie, USA)<br>THP-1 Cat# TIB-202 (ATCC)<br>THP-1 dual cGAS KO (Invivogen)<br>THP-1 dual MAVS KO (Invivogen)<br>THP-1 dual MDA5 KO (Invivogen)<br>THP-1 dual RIG-I KO (Invivogen)<br>THP-1 dual WT (Invivogen)<br>Jurkat Tag Prof. Dr. Oliver Fackler (Heidelberg, Germany)
Authentication	The cell lines were authenticated by ATCC, NIH or their lab of origin and not validated further in our laboratory.
Mycoplasma contamination	Cells were tested routinely to be free of mycoplasma using a PCR based test.
Commonly misidentified lines (See ICLAC register)	No commonly misidentified cell lines were used.

Plants

Seed stocks	N.A.
Novel plant genotypes	N.A.
Authentication	N.A.

Flow Cytometry

Plots

Confirm that:

☒ The axis labels state the marker and fluorochrome used (e.g. CD4-FITC).

☒ The axis scales are clearly visible. Include numbers along axes only for bottom left plot of group (a 'group' is an analysis of identical markers).

☒ All plots are contour plots with outliers or pseudocolor plots.

☒ A numerical value for number of cells or percentage (with statistics) is provided.

Methodology

Sample preparation	MDMs were detached by incubating for 20 min with 1% EDTA at 37°C. To monitor infection levels of MDMs, THP-1 duals cells or PBMCs, around 400,000 cells were harvested, washed in 200 µL of PBS and centrifuged at 1500 rpm for 3 min. Cells were stained with anti-CD4 (PerCP-Cy5.5) with fixable viability dye (780 nm) for 30 min at room temperature. Cells were washed twice with PBS and permeabilized with 200 µL of cytofix/cytoperm permeabilization buffer for 20 min at room temperature. Cells were washed with permwash buffer and incubated in permwash buffer with p24 (PE) antibody for 30 min at room temperature. Cells were washed with permwash buffer and fixed with 2% PFA in PBS. Cells were acquired with BD FACSCanto II Flow Cytometer. To stain intracellular pS3-cofilin levels, anti-pS3 cofilin antibody was added prior to incubation with p24 antibody for 1 hour at 4°C and wash twice with permwash buffer. Anti-rabbit alexa fluor 488 secondary antibody was added together with the p24 antibody. Cells were acquired with BD FACSCanto II Flow Cytometer (BD Biosciences).
Instrument	FACS Calibur or FACS Canto; BD
Software	FlowJo 10.8.1

Cell population abundance	Cell were gated as indicated in the manuscript. Live dead stain was used to exclude dead cells and debris
Gating strategy	all cells SSC-A / FSC-A, single cells FSC-A / FSC-H, alive cells (APC-CY7+), Infected cells (p24 (PE) positive/CD4(PerCP-Cy5.5) negative cells).

☒ Tick this box to confirm that a figure exemplifying the gating strategy is provided in the Supplementary Information.
